# Icariin inhibits hyperglycemia-induced cell death in penile cavernous tissue and improves erectile function in type 1 diabetic rats

**DOI:** 10.1093/sexmed/qfaf017

**Published:** 2025-03-27

**Authors:** Haowei Yang, Wenju Xiong, Jun Jiang, Rui Jiang

**Affiliations:** Department of Urology, the Affiliated Hospital of Southwest Medical University, Luzhou, Sichuan 646000, China; Department of Urology, the Affiliated Hospital of Southwest Medical University, Luzhou, Sichuan 646000, China; Department of Thyroid Surgery; the Affiliated Hospital of Southwest Medical University, Luzhou, Sichuan 646000, China; Department of Urology, the Affiliated Hospital of Southwest Medical University, Luzhou, Sichuan 646000, China

**Keywords:** apoptosis, diabetes, erectile dysfunction, ferroptosis, icariin, pyroptosis, rat

## Abstract

**Background:**

Hyperglycemia can cause endothelial cell (EC) and smooth muscle cell (SMC) death in the penile cavernous tissue of rats and lead to erectile dysfunction (ED).

**Objectives:**

To investigate the proportions of apoptotic, pyroptotic, and ferroptotic cells among ECs and SMCs in the penile cavernous tissue of type 1 diabetic (T1DM) rats and the mechanism by which icariin (ICA) improves the erectile function of T1DM rats.

**Methods:**

A total of 24 9-week-old Sprague–Dawley (SD) rats were randomly divided into 4 groups (*n* = 6): control group, control + ICA group, diabetic mellitus (DM) group, and DM + ICA group. T1DM rats were generated via the intraperitoneal injection of STZ (45 mg/kg). After 8 weeks, the rats in the control + ICA group and the DM + ICA group were administered ICA (10 mg/kg/d) by gavage for 4 weeks. ROS, MDA, SOD, GSH, SM/C, and NO levels, and GPX4, ACSL4, caspase-1, GSDMD, caspase-3, CD31, α-SMA, and p-eNOS/eNOS expression in penile cavernous tissue and the ICPmax/MAP of 21-week-old rats were detected.

**Results:**

The percentage of pyroptotic SMCs in penile cavernosum was no statistically significant difference among these groups. Vs control group, the percentages of apoptotic (20.70% ± 1.60%), pyroptotic (21.02% ± 1.97%), and ferroptotic (9.01% ± 2.00%) ECs and the percentages of apoptotic (15.47% ± 1.36%) and ferroptotic (26.33% ± 3.11%) SMCs in the penile cavernous tissue of the DM group were significantly greater. Vs DM group, the percentages of apoptotic (9.13% ± 1.28%), pyroptotic (13.22 ± 1.26%), and ferroptotic (4.01% ± 0.86%) ECs and the percentages of apoptotic (11.60% ± 1.91%) and ferroptotic (12.71% ± 2.92%) SMCs of the DM + ICA group were significantly lower. Vs the DM group, the levels of caspase-1, GSDMD, ACSL4, and ROS were significantly lower in the penile cavernous tissue of the DM + ICA group. Meanwhile, the levels of GPX4 and maximum intracavernous pressure/mean arterial pressure (ICPmax/MAP) were significantly higher.

**Clinical Implications:**

The combined inhibition of apoptosis, pyroptosis, and ferroptosis in penile cavernous tissue by ICA provides a theoretical basis for the clinical development of multi-target drugs for the treatment of type 1 diabetes-induced ED.

**Strengths and Limitations:**

Further experiments are required to clarify whether other types of cell death are involved in the loss of ECs and SMCs in the penile cavernous tissue of T1DM rats.

**Conclusion:**

Inhibiting oxidative stress and thereby inhibiting apoptosis, pyroptosis, and ferroptosis in ECs and SMCs of penile cavernous tissue constitute one of the mechanisms through which ICA improves erectile function in T1DM rats.

## Introduction

Erectile dysfunction (ED) is more likely to occur in men over 40 years of age and is characterized by the inability to achieve or maintain a full erection sufficient for satisfactory sexual activity for a period of more than 3 months.^1^ Hyperglycemia is one of the most common risk factors for ED.^2^ For male diabetic patients, the incidence of ED is as high as 52.5%.^3^

The pathogenesis of diabetic mellitus erectile dysfunction (DMED) is complex and involves multiple systems, such as endothelial dysfunction, cavernous smooth muscle damage, and changes in hormone levels.^4^ As first-line drugs currently used for the treatment of ED in clinical practice, phosphodiesterase type 5 inhibitors (PDE5is) have an effective rate of only 44% for the treatment of DMED.^5^ The poor therapeutic effect of PDE5is is related to the reduction in the number of endothelial cells (ECs) and smooth muscle cells (SMCs) in the penile cavernous tissue under diabetic conditions.^6^^,^^7^ Increased oxidative stress levels under diabetic conditions represent an important reason for the damage to and death of penile cavernous cells. Antioxidants in the body, such as superoxide dismutase (SOD) and reduced glutathione (GSH), can scavenge reactive oxygen species (ROS). Malondialdehyde (MDA) is the end product of lipid peroxidation and reflects the degree of lipid peroxidation.^8^ A high-glucose environment can cause an increase in ROS production in the penile cavernous tissue, reduced SOD activity and GSH content, and an increase in the MDA content,^9^ which subsequently causes cavernous EC and SMC apoptosis.^9^^,^^10^ Secondary vascular vasomotor dysfunction of the penile cavernous tissue leads to DMED.

However, inhibiting cell apoptosis alone cannot completely improve the erectile function of diabetic rats.^10^^,^^11^ In late-stage DMED rats, the survival rate of ECs in the penile cavernous tissue is only 30%-45%, and the proportion of apoptotic ECs represents less than half of the total lost ECs.^12^ These findings indicate that other forms of cell death may be involved in the death of penile cavernous cells. In addition, studies have shown that pyroptosis and ferroptosis are involved in the development of DMED.^13^^,^^14^

Pyroptosis is a type of proinflammatory programmed cell death that is different from apoptosis. The classical pyroptosis pathway mediated by caspase-1 is currently the most studied pyroptosis pathway.^15^ Caspase-1 and gasdermin-D (GSDMD) are key factors in this process.^16^ Among them, caspase-1 plays a key role in triggering pyroptosis by cleaving GSDMD.^17^ROS promote the formation of the NLRP3 inflammasome, leading to inflammation and pyroptosis.^18^ The penile cavernous tissue of diabetic rat’s exhibits increased expression of pyroptosis-related factors. The classical pyroptosis pathway involving caspase-1 is involved in the loss of ECs in the penile cavernous tissue of diabetic rats and the development of DMED.^12^^,^^13^

Ferroptosis is a type of programmed cell death characterized by iron dependence and ROS-induced lipid peroxidation.^19^ GPX4 is considered a key inhibitory molecule in the classical ferroptosis pathway.^20^ ACSL4, a key protein that promotes ferroptosis, promotes the execution of ferroptosis by catalyzing the synthesis of lipid peroxides.^21^^,^^22^ In in vitro experiments, ROS accumulation triggers ferroptosis in penile cavernous ECs.^23^ The ferroptosis inhibitor FER-1 inhibits ferroptosis in the penile cavernous SMCs of diabetic rats in in vitro experiments.^14^ Increased GPX4 gene expression improves the reduction in the number of penile cavernous ECs and SMCs in diabetic rats.^24^ These findings indicate that ferroptosis is involved in the development of DMED. However, the proportions of apoptotic, pyroptotic, and ferroptotic ECs and SMCs in the penile cavernous tissue of type 1 diabetic (T1DM) rats are currently unknown.

Epimedium is a traditional Chinese herbal medicine that is often used to treat ED. Total flavonoids of Epimedium (TFE) are the main active ingredients of Epimedium.^25^ Icariin (ICA), the most abundant and main active substance in TFE,^26^ is partially metabolized into products such as icariside II (ICSII), icariside I (ICSI), and icaritin (ICT) under the action of the intestinal flora in rats and is subsequently absorbed and utilized.^27^^,^^28^ ICA and its in vivo metabolite ICS II have strong antioxidant capacities.^29^^,^^30^ ICA and ICSII can improve the erectile function of T1DM by reducing the oxidative stress level in the penile cavernous tissue, increasing the expression of nNOS/eNOS, raising the level of cGMP, protecting the ECs and SMCs in the penile cavernous tissue, and increasing the ratio of smooth muscle to collagen.^31-33^ However, the mechanisms of ICA protecting ECs and SMCs are still confusing. ICA and ICSII significantly inhibit the expression of the apoptosis-related proteins, such as Bax and cleaved caspase-3, in the penile cavernous tissue of DMED rats and simultaneously promote the expression of the antiapoptotic factor BCL2, thereby inhibiting the apoptosis of ECs and SMCs in the penile cavernous tissue.^34^^,^^35^ However, there are multiple modes of cell death in penile cavernous tissue of T1DM rats. What are the proportions of apoptotic, pyroptotic, and ferroptotic cells in the ECs and SMCs of the penile cavernous tissue of T1DM rats? Can ICA improve the erectile function of T1DM rats by inhibiting cell pyroptosis and ferroptosis in penile cavernous tissue?

## Materials and methods

### Animals and experiments

In this study, 24 healthy 8-week-old male Sprague–Dawley (SD) rats (all from the SPF-level Animal Experimental Center of Southwest Medical University) were adaptively fed for 1 week and then randomly divided into 4 groups (n = 6): the control group, the control + ICA group, the DM group, and the diabetes + icariin (DM + ICA) group. After fasting for 12 hours, the rats in the DM group and the DM + ICA group were intraperitoneally injected with dissolved streptozotocin (STZ) (45 mg/kg)^36^ and the remaining groups were injected with an equal amount of citrate buffer solution (pH 4.5). After 72 hours, the fasting blood glucose level of diabetic rats was ≥16.7 mmol/L. The body weights and random blood glucose levels of the rats in each group were recorded weekly. Eight weeks after the diabetes model was established, the rats in the DM + ICA group and the control + ICA group were intragastrically administered ICA (10 mg/kg)^26^^,^^37^^,^^38^ (Desite Biotechnology, purity ≥98%) every day, and the remaining rats were intragastrically administered an equal amount of solvent without ICA (2% DMSO +98% normal saline)^26^^,^^38^^,^^39^ every day for 4 weeks.^37^^,^^40^ All experimental procedures were performed in accordance with the *Guide for the Care and Use of Laboratory Animals* (NIH Publication No. 85-23; revised in 1996) and approved by the Animal Management and Use Committee of Southwest Medical University (SWMU20240059).

### Maximum intracavernous pressure/mean arterial pressure (ICPmax/MAP)

The rats were anesthetized via an intraperitoneal injection of 1% pentobarbital sodium (40 mg/kg). Prefilled heparin 24G and 26G indwelling needles were placed in the left common carotid artery and penile cavernous tissue, respectively. A pressure sensor was connected to measure the MAP and ICPmax. Electrical stimulation was applied to the penile cavernous nerve (intensity: 3 V/5 V; frequency: 12 Hz; amplitude: 5 ms; duration: 30 s; interval: 3 minutes).^12^ ICP and MAP were recorded using the BL-420S biological signal acquisition system (TECHMAN Technology, Chengdu, China). Then, the rats were sacrificed by excessive anesthesia. Carotid artery blood was collected. Serum testosterone was detected according to the instructions of the rat serum testosterone ELISA kit (Elabscience Biotechnology, Wuhan, China; E-OSEL-R0003). The penis of each rat was collected. The penile cavernous tissue was divided into five sections from the front to the back and used for paraffin embedding (Masson and Prussian blue staining), frozen embedding (ROS and immunofluorescence assessments), ELISA, flow cytometry, and Western blot experiments in sequence.

### Detection of NO, MDA, SOD, GSH/GSSG, and GSH levels in the penile cavernous tissue of the rats

Fresh penile cavernous tissue was rapidly frozen in liquid nitrogen and then ground into powder. Subsequently, RIPA lysis buffer (Beyotime Biotechnology, Shanghai, China; P0013B) was added at a ratio of 1 mg: 7.5 μL, and the mixture was thoroughly lysed for 1.5 hours. After that, the sample was centrifuged, and the supernatant was collected. The total protein concentration was detected using colorimetry with the BCA protein assay kit (Beyotime; P0012S). Then, according to the instructions of the corresponding kits for NO (Beyotime; S0021S), MDA (Beyotime; S0131S), SOD(Beyotime; S0101S), and GSH/GSSG(Beyotime; S0053), the corresponding reagents and test samples were added to a 96-well plate. After the incubation was complete, the absorbance at the corresponding wavelength was detected using a microplate reader. A standard curve was established and the content of the measured index in the sample was calculated based on the formula provided with the kit in combination with the standard curve.

### Detection of ROS levels in the penile cavernous tissue of rats

Fresh penile cavernous tissue was embedded in optimal cutting temperature (OCT) compound and then made into frozen sections (5 μm). The sections were fixed in 10% neutral formalin for 10 minutes. In accordance with the instructions of the ROS detection kit (Beyotime; S0033S), the DCFH-DA probe was diluted with 10% goat serum at a ratio of 1:1000. Appropriate amounts of DCFH-DA probe were placed drop-wise onto a glass slide and incubated in a 37°C incubator for 30 minutes. After being sufficiently washed, the nuclei were stained with DAPI. Then, the glass slides were observed under a fluorescence microscope. After digital images were obtained, the images were analyzed via ImageJ v1.8.0 (National Institutes of Health, USA).

### Masson staining and Prussian blue staining

Paraffin-embedded sections of penile cavernous tissue (5 μm) were stained according to the instructions of the Masson trichrome staining kit (Solarbio, Beijing, China; G1340). Another section was stained according to the instructions of the Prussian blue staining kit (Solarbio; G1422). The sections were subsequently scanned with a digital slice scanner. After the images were obtained, Image-Pro Plus 6.0 (Media Cybernetics, USA) was used to analyze the images.

### Immunofluorescence

The frozen sections of the penile cavernous tissue fixed with 10% neutral formalin were blocked with 10% goat serum for 30 minutes. The following primary antibodies were subsequently added as needed and incubated overnight at 4°C: caspase-1 p20 monoclonal antibody (1:100) (Santa Cruz, Texas, USA; sc-398 715), ACSL4 monoclonal antibody (1:100) (Proteintech, Wuhan, China; 66 617-1-Ig), CD31 monoclonal antibody (1:75) (Abcam, UK; ab182981), and α-SMA monoclonal antibody (1:200) (Abcam; ab124964). After the sections were washed, the following fluorescent secondary antibodies were added: goat anti-mouse IgG DyLight 594 (1:200) and goat anti-rabbit IgG DyLight 488 (1:200) (Abbkine Scientific, Georgia, US). After being incubated in the dark at 37 °C for 30 minutes, a DAPI solution was added for 10 minutes to stain the nuclei. After the sections were washed and mounted, digital images were obtained by scanning the sections with a fluorescence microscope. The images were subsequently analyzed via ImageJ v1.8.0 (NIH, USA).

### Flow cytometry

Fresh penile cavernous tissue was fully minced in PBS containing penicillin and streptomycin and then fully digested with 1% collagenase II for 2 hours to obtain a tissue suspension. Subsequently, a serum-containing medium was added to terminate the reaction. After being filtered through a 200-mesh cell filter, the supernatant was discarded by centrifugation, and the cells were resuspended in the complete medium. The cells were fixed with 4% formaldehyde for 10 minutes, washed, and precooled. 70% ethanol was added for permeabilization for 30 minutes. Then, the cells were washed and resuspended. The following antibodies were added to the samples as needed and incubated at 4 °C in the dark for 30 minutes: PE-conjugated anti-rat CD31 (BD Biosciences, USA; 555 027), PE-conjugated anti-α-SMA (Abcam; ab208844), AlexaFluor647-conjugated anti-caspase-1 (Santa Cruz; sc-398 715), FITC-conjugated anti-ACSL4 (Abcam; ab155282), and AlexaFluor488-conjugated anti-cleaved caspase-3 (Cell Signaling Technology, UK; 9669S) antibodies. The cells were resuspended after the unbound antibodies were washed off. The fluorescence compensation was adjusted using a flow cytometer, and the expression rates of CD31, α-SMA, caspase-3, caspase-1, and ACSL4 in penile cavernous cells were detected. The data were analyzed via FlowJo v10.4 (Becton Dickinson Biosciences, USA). The rate of apoptotic/pyroptotic/ferroptotic cells in ECs was calculated as the number of CD31 and (caspase-3/caspase-1/ACSL4) double-positive cells/the number of total CD31 positive cells. The rate of apoptotic/pyroptotic/ferroptotic cells in SMCs was calculated as the number of α-SMA and (caspase-3/caspase-1/ACSL4) double-positive cells/the number of total α-SMA positive cells.^12^^,^^21^^,^^41^

### Western blot

After the penile cavernous tissue was fully lysed to obtain proteins, the supernatant was collected, and the total protein concentration was determined by the BCA method. Then, a 1/4 volume of protein loading buffer (5X, Beyotime) was added, and the mixture was denatured at 98 °C for 10 minutes and stored at -20 °C for subsequent Western blot experiments. A 7.5% or 12.5% SDS–polyacrylamide gel was prepared according to the instructions, and electrophoresis was performed using the samples. After electrophoresis, the proteins were transferred to a polyvinylidene fluoride (PVDF) membrane. After transfer, the samples were incubated with a rapid blocking buffer (Epizyme, Shanghai, China) at room temperature for 15 minutes. After blocking, the following primary antibodies were added as needed and incubated overnight at 4 °C: caspase-1 p20 monoclonal antibody (1:1500) (Santa Cruz; sc-398 715), GSDMD monoclonal antibody (1:1500) (Santa Cruz; sc-393 581), ACSL4 monoclonal antibody (1:5000) (Abcam; ab155282), GPX4 monoclonal antibody (1:1000) (Abcam; ab125066), eNOS monoclonal antibody (1:2000) (Abcam; ab300071), p-eNOS monoclonal antibody (1:2000) (Cell Signaling Technology; 9571S), and β-actin monoclonal antibody (1:2000) (Abcam; ab6276). After the membranes were washed, the corresponding secondary antibodies were added, and the samples were incubated at room temperature for 2 hours. After the membrane was washed, an enhanced chemiluminescence (ECL) solution was used to develop the membrane, and images were obtained. After the target protein was developed, the membrane was washed, antibody stripping buffer (Epizyme, Shanghai, China) was added, and the membrane was incubated on a shaker for 15 minutes. Then, the above blocking steps were repeated to develop the internal reference. ImageJ v1.8.0 (NIH, USA) was used to perform data analysis on the obtained images.

### Data analysis

Statistical processing was performed via GraphPad Prism-9.5 (GraphPad Software, San Diego, CA, USA) software. The Shapiro–Wilk test was used to test the normality of the data. The Brown-Forsythe test or Bartlett test was used to analyze the data. The results are expressed as $\overline{\mathrm{x}}\pm \mathrm{SD}$. One-way ANOVA was used for comparisons between groups. Tukey’s test was used for post hoc testing. A difference was considered statistically significant when *P* ≤ .05.

## Results

### Effects of ICA on random blood glucose, body weight, and serum testosterone levels in rats

At 9 weeks of age, no statistically significant difference in the initial body weight or blood glucose level was noted among the groups of rats. At 21 weeks of age, no statistically significant difference in body weight or testosterone levels was noted among the groups of rats. At 21 weeks, the blood glucose levels of the rats in the DM group (21.22 ± 2.11 mmol/L) were significantly greater compared with the control group (6.34 ± 0.61 mmol/L) (*P* < .05), and no significant difference in blood glucose levels was noted between the DM + ICA group (19.98 ± 1.56 mmol/L) and the DM group (21.22 ± 2.11 mmol/L) (Supplementary Table 1).

### Icariin improves erectile function in T1DM rats

Under 5 V electrical stimulation, the ICPmax/MAP of the rats in the DM group (29.60% ± 2.40%) was significantly lower than that in the control group (70.03% ± 2.63%) (*P* < .05). The ICPmax/MAP of the rats in the DM + ICA group (54.52% ± 2.82%) was significantly greater than that of the DM group (*P* < .05) but was still significantly lower than that of the control + ICA group (72.95% ± 3.46%) (*P* < .05) (Supplementary Figure 1).

### Icariin improves oxidative stress in the penile cavernous tissue of T1DM rats

**Figure 1:**
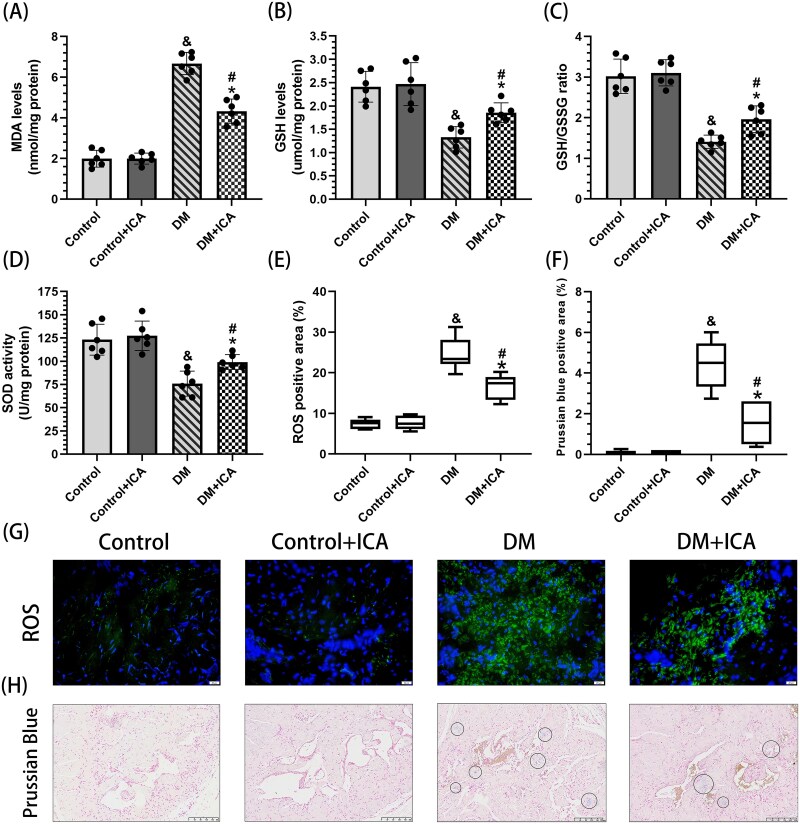
Effect of icariin on oxidative stress in the penile cavernous tissue of DM rats. (A) MDA levels, (B) GSH levels, (C) the GSH/GSSG ratio, and (D) SOD activity in each group were measured via ELISA. (E) Semiquantitative analysis and (G) representative images (40×) of ROS in each group. (F) Semiquantitative analysis and (H) representative images (15×) of Prussian blue staining showing nucleus (red areas) and iron deposition (blue areas). And *P* < .05 vs the control group. ^*^P < .05 vs the control + ICA group. ^#^*P* < .05 vs the DM group.

In the penile cavernous tissue of rats in the DM group, the area positive for ROS (24.62% ± 4.02%) (Figure 1E, G) and the content of MDA (6.67 ± 0.54 nmol/mg prot) (Figure 1A) were significantly greater than those in the control group (p < 0.05), while the activity of SOD (75.88 ± 13.53 U/mg prot), the content of GSH (1.32 ± 0.23 μmol/mg prot), and the GSH/GSSG ratio (Figure 1B-D) were significantly lower than those in the control group (*P* < .05). In the penile cavernous tissue of rats in the DM + ICA group, the area positive for ROS (16.59% ± 3.06%) and the content of MDA (4.33 ± 0.59 nmol/mg prot) were significantly lower than those in the DM group (*P* < .05), while the activity of SOD (75.88 ± 13.53 U/mg prot), the content of GSH (1.32 ± 0.23 μmol/mg prot), and the GSH/GSSG ratio were significantly higher than those in the DM group (*P* < .05).

### Icariin inhibits EC pyroptosis in the penile cavernous tissue of T1DM rats

**Figure 2:**
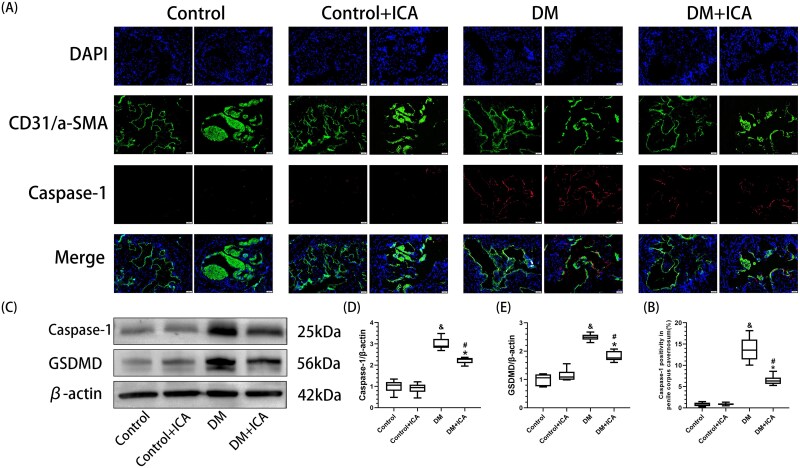
Effect of icariin on pyroptosis in the penile cavernous tissue of DM rats. (A) Representative images (20×) of the expression of caspase-1 in the penile cavernous tissue of the rats in each group, as determined by an immunofluorescence assay. The blue fluorescence represents DAPI. Green fluorescence represents CD31 (L) or α-SMA (R). Red fluorescence represents caspase-1. (B) Semiquantitative analysis of the percentage of caspase-1-positive penile cavernous tissue in each group. (C) Western blot analysis of the expression of caspase-1 and GSDMD in the penile cavernous tissue of each group. (D, E) Semiquantitative analysis of caspase-1 and GSDMD in penile cavernous tissue from each group. & *P* < .05 vs the control group. ^*^P < .05 vs the control + ICA group. ^#^*P* < .05 vs the DM group.

Compared with those in the control group, the expression levels of caspase-1 and GSDMD in the penile cavernous tissue of the rats in the DM group were significantly greater (*P* < .05) (Figure 2C-E). Compared with that in the control group, the positive area of caspase-1 in the penile cavernous tissue of the rats in the DM group (13.63% ± 2.71%) was significantly greater (*P* < .05) (Figure 2B), and caspase-1 was expressed mainly in ECs [CD31(+) and caspase-1 (+)]. Moreover, no obvious α-SMA or caspase-1 double-positive cells were observed (Figure 2A). Compared with the DM group, caspase-1 and GSDMD expression and the positive area of caspase-1 (6.49% ± 0.95%) in the penile cavernous tissue of the rats in the DM + ICA group were significantly lower (*P* < .05).

### Icariin inhibits EC and smooth muscle cell ferroptosis in the penile cavernous tissue of T1DM rats

**Figure 3:**
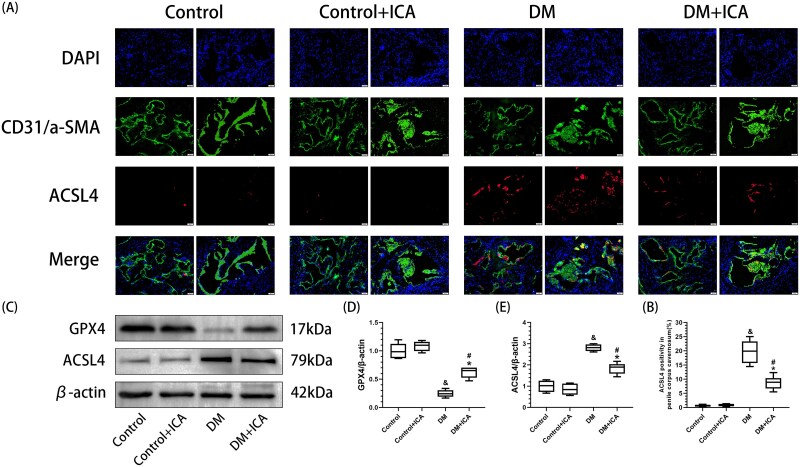
Effect of icariin on ferroptosis in the penile cavernous tissue of DM rats. (A) Representative images (20×) of the expression of ACSL4 in the penile cavernous tissue of the rats in each group, as determined by an immunofluorescence assay. The blue fluorescence represents DAPI. Green fluorescence represents CD31 (L) or α-SMA (R). Red fluorescence represents ACSL4. (B) Semiquantitative analysis of the percentage of ACSL4-positive penile cavernous tissue in each group. (C) GPX4 and ACSL4 expression in the penile cavernous tissue of the rats in each group was measured by western blotting. (D, E) Semiquantitative analysis of GPX4 and ACSL4 expression in penile cavernous tissue from each group. And *P* < .05 vs the control group. ^*^P < .05 vs the control + ICA group. ^#^*P* < .05 vs the DM group.

In the DM group, ACSL4 expression in the penile cavernous tissue of the rats (Figure 3C, E) and the positive area of iron-stained foci (4.42% ± 1.16%) (Figure 1H, F) were significantly greater than those in the control group (*P* < .05), and GPX4 expression was significantly lower than that in the control group (Figure 3C, D). Compared with that in the control group, the area of ACSL4-positive penile cavernous tissue in the DM group (19.64% ± 3.73%) was significantly greater (*P* < .05) (Figure 3B), and ACSL4 was expressed mainly in SMCs [α-SMA(+) and ACSL4(+)] and ECs [CD31(+) and ACSL4(+)] (Figure 3A). Compared with that in the DM group, GPX4 expression in the penile cavernous tissue of the rats in the DM + ICA group was significantly greater. In addition, ACSL4 expression, the positive area of iron-stained foci (4.42% ± 1.16%), and the positive area of ACSL4 (8.67% ± 2.26%) were significantly lower in the DM + ICA group than in the DM group.

### Proportions of apoptotic, pyroptotic, and ferroptotic endothelial cells in the penile cavernous tissue of T1DM rats

**Figure 4:**
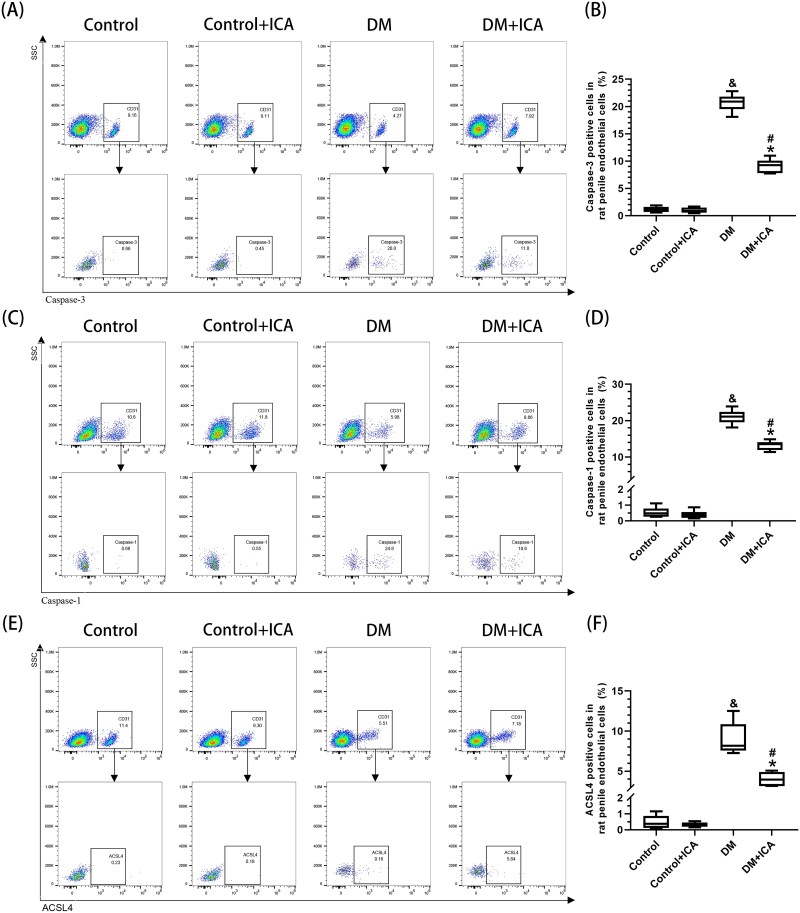
Proportion of different modes of endothelial cell (EC) death in the penile cavernous tissue of the rats. (A,C,E) Representative images of the proportion of ECs among total penile cavernous cells and the proportions of (A) apoptotic, (C) pyroptotic, and (E) ferroptotic cells among the ECs in each group, as determined by flow cytometry. (B, D, F) Data are presented as the proportions of (B) apoptotic, (D) pyroptotic, and (F) ferroptotic cells among the penile cavernosum ECs in each group. And *P* < .05 vs the control group. ^*^P < .05 vs the control + ICA group. ^#^*P* < .05 vs the DM group.

The percentages of apoptotic cells (20.70% ± 1.60%) (Figure 4A, B), pyroptotic cells (21.02% ± 1.97%) (Figure 4C, D), and ferroptotic cells (9.01% ± 2.00%) (Figure 4E, F) among the ECs in the penile cavernous tissue of the rats in the DM group were significantly greater than those in the control group (*P* < .05). The percentages of apoptotic cells (9.13% ± 1.28%), pyroptotic cells (13.22% ± 1.26%), and ferroptotic cells (4.01% ± 0.86%) among the ECs in the penile cavernous tissue of the rats in the DM + ICA group were significantly lower than those in the DM group (*P* < .05) but still significantly greater than those observed in the control + ICA group (*P* < .05).

### Proportions of apoptotic, pyroptotic, and ferroptotic SMCs in the penile cavernous tissue of T1DM rats

**Figure 5:**
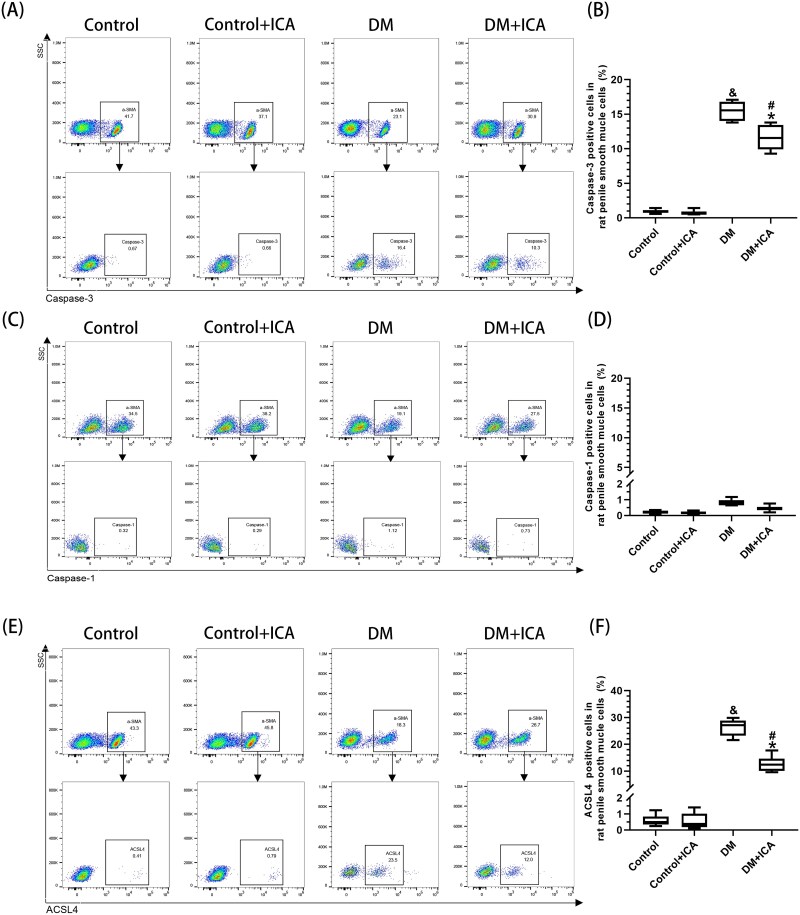
The proportions of different modes of smooth muscle cell (SMC) death in the penile cavernous tissue of the rats. (A,C,E) Representative images of the proportion of SMCs among total penile cavernous cells and the proportions of (A) apoptotic, (C) pyroptotic, and (E) ferroptotic SMCs in each group, as determined by flow cytometry. (B, D, F) Data are presented as the proportions of (B) apoptotic, (D) pyroptotic, and (F) ferroptotic cells among the penile cavernosum SMCs in each group. And *P* < .05 vs the control group. ^*^P < .05 vs the control + ICA group. ^#^*P* < .05 vs the DM group.

The percentages of pyroptotic penile cavernosum SMCs were not statistically different among all the groups (Figure 5C, D). The percentages of apoptotic cells (15.47% ± 1.36%) (Figure 5A, B) and ferroptotic cells (26.33% ± 3.11%) (Figure 5E, F) among SMCs in the penile cavernous tissue of rats in the DM group were significantly greater than those observed in the control group (*P* < .05). The percentages of apoptotic cells (11.60% ± 1.91%) and ferroptotic cells (12.71% ± 2.92%) among SMCs in the penile cavernous tissue of rats in the DM + ICA group were significantly lower than those noted in the DM group (*P* < .05) but still significantly greater than those in the control + ICA group (*P* < .05).

### Icariin improves EC function and inhibits SMC fibrosis in the penile cavernous tissue of diabetic rats

**Figure 6:**
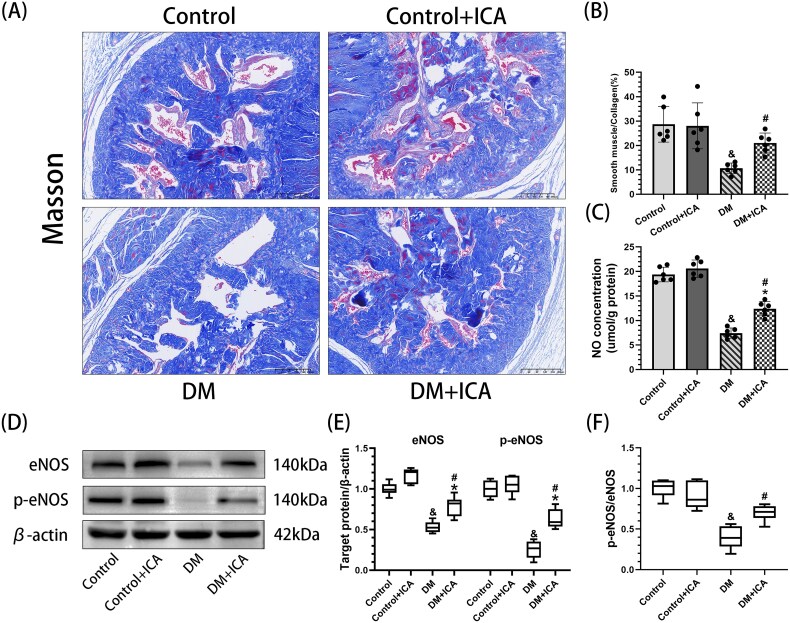
Effect of icariin on the fibrosis of penile cavernous tissue in DM rats. (A) Representative images (15×) of Masson's trichrome staining showing smooth muscle cells (SMCs) and collagen fibers. (B) Semiquantitative analysis of the SM/C ratio of each group. (C) NO levels in each group were measured using ELISA. (D) Western blot analysis of the expression of eNOS and peNOS in penile cavernous tissue from each group. (E, F) Semiquantitative analysis of eNOS and p-eNOS levels and the p-eNOS/eNOS ratio in penile cavernous tissue from each group. And *P* < .05 vs the control group. ^*^P < .05 vs the control + ICA group. ^#^*P* < .05 vs the DM group.

Compared with those in the control group, the ratios of p-eNOS to eNOS and the contents of NO (7.42 ± 1.04 μmol/g prot) (Figure 6C-F) and SM/C (10.63% ± 2.17%) (Figure 6A, B) in penile cavernous tissue were significantly lower in the DM group (*P* < .05). Compared with those in the diabetic group, the ratio of p-eNOS to eNOS, NO content (12.41 ± 1.45 μmol/g prot), and the SM/C ratio (21.03% ± 4.07%) in penile cavernous tissue of rats in the DM + ICA were significantly greater (*P* < .05).

## Discussion

In this study, ROS levels in the penile cavernous tissue of rats in the DM group were significantly higher than those in the control group. These results are consistent with those of previous studies.^24^ The rats in the DM + ICA group exhibited higher SOD activity and a higher GSH/GSSG ratio as well as lower MDA and ROS levels in the penile cavernous tissue compared with rats in the DM group. These findings suggest that ICA treatment can effectively inhibit the level of oxidative stress in the penile cavernous tissue under T1DM conditions.

The exacerbation of oxidative stress can cause apoptosis, pyroptosis, and ferroptosis in the penile cavernous tissue of rats.^9^^,^^18^^,^^19^ This study reports for the first time the proportions of apoptotic, pyroptotic, and ferroptotic cells among ECs and SMCs in the penile cavernous tissue of T1DM rats before and after ICA treatment (Figures 4 and 5). After ICA treatment, the percentages of apoptotic ECs and SMCs in the penile cavernous tissue of the rats in the DM + ICA group were significantly lower than those in the DM group. These findings are consistent with the results of previous studies.^31^^,^^32^ In addition, ICA treatment inhibited pyroptosis and ferroptosis in the penile cavernous tissue of DM rats. After ICA treatment, the percentages of pyroptotic and ferroptotic ECs and the percentage of ferroptotic SMCs in the penile cavernous tissue of the rats in the DM + ICA group were significantly lower than those in the DM group. Moreover, this study found that pyroptosis mainly occurred in ECs in the penile cavernous tissue of T1DM rats, while no obvious pyroptosis was observed in SMCs. This indicates that the modes of cell death in different types of cells in the penile cavernous tissue of T1DM rats are different. It suggests that different treatments of inhibiting cell death can be adopted according to different types of cells to maximize the reduction of the loss of penile cavernous cells in T1DM rats.

Therefore, under hyperglycemic conditions, apoptosis, pyroptosis, and ferroptosis are involved in the loss of ECs and SMCs in the penile cavernous tissue of rats to varying degrees. ICA inhibits the apoptosis, pyroptosis, and ferroptosis of ECs and SMCs by reducing oxidative stress in the penile cavernous tissue of T1DM rats, diminishing the loss of the number of ECs and SMCs. On the one hand, it subsequently improves EC function, and the ratio of p-eNOS/eNOS and the NO content in the penile cavernous tissue of DM rats increase significantly. On the other hand, ICA inhibits smooth muscle fibrosis, and the SM/C in the penile cavernous tissue of DM rats increases significantly. Eventually, the ICPmax/MAP of DM rats increases significantly, and erectile function improves.

However, programmed cell death of ECs and SMCs in penile cavernous tissue under hyperglycemic conditions may involve other types in addition to apoptosis, pyroptosis, and ferroptosis. Necroptosis is a form of programmed cell death that is different from apoptosis, pyroptosis, and ferroptosis. Its morphology is similar to that of necrotic cells.^42^ PANoptosis is a highly coordinated and dynamically balanced mode of programmed inflammatory cell death that combines the main features of pyroptosis, apoptosis, and necroptosis, and its morphology shows a mixture of characteristics of various modes of cell death.^43^ The regulation of PANoptosis is closely related to the apoptosis and pyroptosis signaling pathways.^43^ Therefore, whether type 1 diabetes may cause necroptosis and PANoptosis in penile cavernous cells and the effect of ICA on these processes requires further study.

## Conclusion

Inhibiting oxidative stress and thereby inhibiting apoptosis, pyroptosis, and ferroptosis in ECs and SMCs of penile cavernous tissue is one of the mechanisms for ICA improving the erectile function of T1DM rats.

## Supplementary Material

Supplementary_Figure_qfaf017

Supplementary_Table_qfaf017

Supplementary_Figure_qfaf017

## References

[1] Shindel AW, Lue TF. Managing sexual dysfunction in 2021 and beyond. Urol Clin North Am. 2021;48(4):xv–xvi. 10.1016/j.ucl.2021.07.004 34602182

[2] Defeudis G, Mazzilli R, Tenuta M, et al. Erectile dysfunction and diabetes: a melting pot of circumstances and treatments. Diabetes Metab Res Rev. 2022;38(2):e3494. 10.1002/dmrr.3494 34514697 PMC9286480

[3] Shamloul R, Ghanem H. Erectile dysfunction. Lancet. 2013; 381(9861):153–165. 10.1016/S0140-6736(12)60520-0 23040455

[4] Castela Â, Costa C. Molecular mechanisms associated with diabetic endothelial-erectile dysfunction. Nat Rev Urol. 2016; 13(5):266–274. 10.1038/nrurol.2016.23 26878803

[5] Liu DF, Jiang H, Hong K, Zhao LM, Tang WH, Ma LL. Influence of erectile dysfunction course on its progress and efficacy of treatment with phosphodiesterase type 5 inhibitors. Chin Med J. 2010;123(22):3258–3261 21163126

[6] de Souza ILL, Ferreira EDS, Vasconcelos LHC, Cavalcante FA, da Silva BA. Erectile dysfunction: key role of cavernous smooth muscle cells. Front Pharmacol. 2022;13:895044. 10.3389/fphar.2022.895044 35865945 PMC9294450

[7] Traish AM, Galoosian A. Androgens modulate endothelial function and endothelial progenitor cells in erectile physiology. Korean J Urol. 2013;54(11):721–731. 10.4111/kju.2013.54.11.721 24255752 PMC3830963

[8] Singh A, Kukreti R, Saso L, Kukreti S. Mechanistic insight into oxidative stress-triggered signaling pathways and type 2 diabetes. Molecules. 2022;27(3):950. 10.3390/molecules27030950 35164215 PMC8840622

[9] Li Z, Jia B, Guo Z, et al. Therapeutic potential of salidroside in type 1 diabetic erectile dysfunction: attenuation of oxidative stress and apoptosis via the Nrf2/HO-1 pathway. PLoS One. 2024;19(7):e0306926. 10.1371/journal.pone.0306926 38990890 PMC11238988

[10] Zhang Z, Zhang HY, Zhang Y, Li H. Correction to: inactivation of the Ras/MAPK/PPARγ signaling axis alleviates diabetic mellitus-induced erectile dysfunction through suppression of corpus cavernosal endothelial cell apoptosis by inhibiting HMGCS2 expression. Endocrine. 2021;71(2):532–533. 10.1007/s12020-020-02561-5 33394387

[11] Liu K, Cui K, Feng H, et al. JTE-013 supplementation improves erectile dysfunction in rats with streptozotocin-induced type I diabetes through the inhibition of the rho-kinase pathway, fibrosis, and apoptosis. Andrology. 2020;8(2):497–508. 10.1111/andr.12716 31610097

[12] Li J, Jiang Q, Jiang J, Jiang R. Mode of cell death in the penile cavernous tissue of type 1 diabetes mellitus rats. J Sex Med. 2024;21(8):652–662. 10.1093/jsxmed/qdae067 38972660

[13] Luo C, Peng Y, Zhou X, et al. NLRP3 downregulation enhances engraftment and functionality of adipose-derived stem cells to alleviate erectile dysfunction in diabetic rats. Front Endocrinol (Lausanne). 2022;13:913296. 10.3389/fendo.2022.913296 35937790 PMC9354456

[14] Xu W, Sun T, Wang J, et al. Ferroptosis is involved in corpus cavernosum smooth muscle cells impairment in diabetes mellitus-induced erectile dysfunction. Andrology. 2023;11(2):332–343. 10.1111/andr.13291 36098277 PMC10087266

[15] Liu P, Lu Z, Liu L, et al. NOD-like receptor signaling in inflammation-associated cancers: from functions to targeted therapies. Phytomedicine. 2019;64:152925. 10.1016/j.phymed.2019.152925 31465982

[16] Shi J, Gao W, Shao F. Pyroptosis: Gasdermin-mediated programmed necrotic cell death. Trends Biochem Sci. 2017;42(4):245–254. 10.1016/j.tibs.2016.10.004 27932073

[17] Yu P, Zhang X, Liu N, Tang L, Peng C, Chen X. Pyroptosis: mechanisms and diseases. Signal Transduct Target Ther. 2021;6(1):128. 10.1038/s41392-021-00507-5 33776057 PMC8005494

[18] Kelley N, Jeltema D, Duan Y, He Y. The NLRP3 inflammasome: an overview of mechanisms of activation and regulation. Int J Mol Sci. 2019;20(13):3328. 10.3390/ijms20133328 31284572 PMC6651423

[19] Jiang X, Stockwell BR, Conrad M. Ferroptosis: mechanisms, biology and role in disease. Nat Rev Mol Cell Biol. 2021;22(4):266–282. 10.1038/s41580-020-00324-8 33495651 PMC8142022

[20] Ingold I, Berndt C, Schmitt S, et al. Selenium utilization by GPX4 is required to prevent hydroperoxide-induced ferroptosis. Cell. 2018;172(3):409–422.e21. 10.1016/j.cell.2017.11.048 29290465

[21] Doll S, Proneth B, Tyurina YY, et al. ACSL4 dictates ferroptosis sensitivity by shaping cellular lipid composition. Nat Chem Biol. 2017;13(1):91–98. 10.1038/nchembio.2239 27842070 PMC5610546

[22] Ding K, Liu C, Li L, et al. Acyl-CoA synthase ACSL4: an essential target in ferroptosis and fatty acid metabolism. Chin Med J. 2023;136(21):2521–2537. 10.1097/CM9.0000000000002533 37442770 PMC10617883

[23] Shi HX, Zhao X, Yang H, Cheng Y, Jiang J, Jiang R. Low androgen levels induce ferroptosis of rat penile cavernous endothelial cells. Sex Med. 2023;11(4):1–8. 10.1093/sexmed/qfad043 PMC10401903 37547873

[24] Xu W, Sun T, Wang J, et al. GPX4 alleviates diabetes mellitus-induced erectile dysfunction by inhibiting Ferroptosis. Antioxidants (Basel). 2022;11(10):1896. 10.3390/antiox11101896 36290619 PMC9598206

[25] Ma H, He X, Yang Y, Li M, Hao D, Jia Z. The genus Epimedium: an ethnopharmacological and phytochemical review. J Ethnopharmacol. 2011;134(3):519–541. 10.1016/j.jep.2011.01.001 21215308

[26] Makarova MN, Pozharitskaya ON, Shikov AN, Tesakova SV, Makarov VG, Tikhonov VP. Effect of lipid-based suspension of Epimedium koreanum Nakai extract on sexual behavior in rats. J Ethnopharmacol. 2007;114(3):412–416. 10.1016/j.jep.2007.08.021 17890032

[27] Xu W, Zhang Y, Yang M, et al. LC-MS/MS method for the simultaneous determination of icariin and its major metabolites in rat plasma. J Pharm Biomed Anal. 2007;45(4):667–672. 10.1016/j.jpba.2007.07.007 17706393

[28] Liu J, Lou YJ. Determination of icariin and metabolites in rat serum by capillary zone electrophoresis: rat pharmacokinetic studies after administration of icariin. J Pharm Biomed Anal. 2004;36(2):365–370. 10.1016/j.jpba.2004.06.021 15496330

[29] Wang X, Wang J, Tian L. Icariin ameliorates TNF-α/IFN-γ-induced oxidative stress, inflammatory response and apoptosis of human immortalized epidermal cells through the WTAP/SERPINB4 axis. Arch Dermatol Res. 2024;316(8):557. 10.1007/s00403-024-03281-w 39177922

[30] Zhang J, Li S, Zhang S, et al. Effect of Icariside II and metformin on penile erectile function, histological structure, mitochondrial autophagy, glucose-lipid metabolism, angiotensin II and sex hormone in type 2 diabetic rats with erectile dysfunction. Sex Med. 2020;8(2):168–177. 10.1016/j.esxm.2020.01.006 32147433 PMC7261708

[31] Zhou F, Xin H, Liu T, et al. Effects of icariside II on improving erectile function in rats with streptozotocin-induced diabetes. J Androl. 2012;33(5):832–844. 10.2164/jandrol.111.015172 22403279

[32] Liu T, Xin H, Li WR, et al. Effects of icariin on improving erectile function in streptozotocin-induced diabetic rats. J Sex Med. 2011;8(10):2761–2772. 10.1111/j.1743-6109.2011.02421.x 21967314

[33] Zhang J, Wang YB, Ma CG, et al. Icarisid II, a PDE5 inhibitor from Epimedium wanshanense, increases cellular cGMP by enhancing NOS in diabetic ED rats corpus cavernosum tissue. Andrologia. 2012;44(Suppl 1):87–93. 10.1111/j.1439-0272.2010.01144.x 21729132

[34] Wang X, Liu C, Xu Y, et al. Combination of mesenchymal stem cell injection with icariin for the treatment of diabetes-associated erectile dysfunction. PLoS One. 2017;12(3):e0174145. 10.1371/journal.pone.0174145 28350842 PMC5369760

[35] Wang L, Xu Y, Li H, et al. Antioxidant icariside II combined with insulin restores erectile function in streptozotocin-induced type 1 diabetic rats. J Cell Mol Med. 2015;19(5):960–969. 10.1111/jcmm.12480 25781208 PMC4420599

[36] Furman BL . Streptozotocin-induced diabetic models in mice and rats. Curr Protoc. 2021;1(4):e78. 10.1002/cpz1.78 33905609

[37] Liu QW, Yang ZH, Jiang J, Jiang R. Icariin modulates eNOS activity via effect on post-translational protein-protein interactions to improve erectile function of spontaneously hypertensive rats. Andrology. 2021;9(1):342–351. 10.1111/andr.12875 33507631

[38] Shindel AW, Xin ZC, Lin G, et al. Erectogenic and neurotrophic effects of icariin, a purified extract of horny goat weed (Epimedium spp.) in vitro and in vivo. J Sex Med. 2010;7(4_Part_1):1518–1528. 10.1111/j.1743-6109.2009.01699.x 20141584 PMC3551978

[39] Li P, Zhao L. Developing early formulations: practice and perspective. Int J Pharm. 2007;341(1-2):1–19. 10.1016/j.ijpharm.2007.05.049 17658228

[40] Xu Y, Xin H, Wu Y, et al. Effect of icariin in combination with daily sildenafil on penile atrophy and erectile dysfunction in a rat model of bilateral cavernous nerves injury. Andrology. 2017;5(3):598–605. 10.1111/andr.12341 28296277

[41] Doitsh G, Galloway NL, Geng X, et al. Cell death by pyroptosis drives CD4 T-cell depletion in HIV-1 infection. Nature. 2017;544(7648):124. 10.1038/nature22066 28329768

[42] Ai Y, Meng Y, Yan B, Zhou Q, Wang X. The biochemical pathways of apoptotic, necroptotic, pyroptotic, and ferroptotic cell death. Mol Cell. 2024;84(1):170–179. 10.1016/j.molcel.2023.11.040 38181758

[43] Wang Y, Kanneganti TD. From pyroptosis, apoptosis and necroptosis to PANoptosis: a mechanistic compendium of programmed cell death pathways. Comput Struct Biotechnol J. 2021;19(1):4641–4657. 10.1016/j.csbj.2021.07.038 34504660 PMC8405902

