# Supplementary figures and images for: Icariin inhibits hyperglycemia-induced cell death in penile cavernous tissue and improves erectile function in type 1 diabetic rats
Source: Sex Med. 2025 Mar 27;13(1):qfaf017. doi: 10.1093/sexmed/qfaf017 (PMC11950537; doi:10.1093/sexmed/qfaf017)

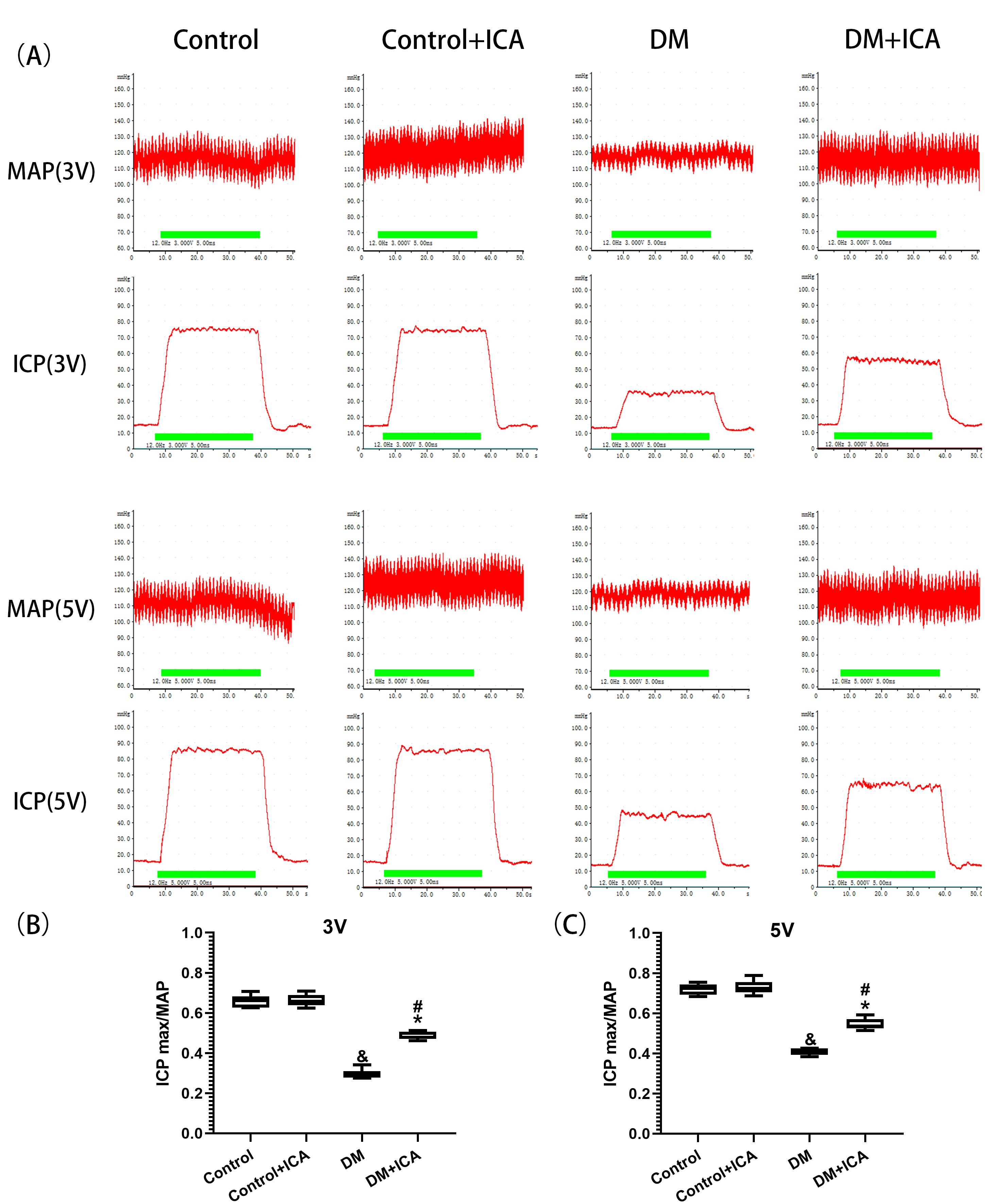

Supplement: Supplementary_Figure_qfaf017 [file supplementary_figure_qfaf017.jpeg]
