# Supplementary material for: Icariin inhibits hyperglycemia-induced cell death in penile cavernous tissue and improves erectile function in type 1 diabetic rats
Source: Sex Med. 2025 Mar 27;13(1):qfaf017. doi: 10.1093/sexmed/qfaf017 (PMC11950537; doi:10.1093/sexmed/qfaf017)
Supplement: Supplementary_Table_qfaf017 [file supplementary_table_qfaf017.docx]

Supplementary table: Body weight, Blood glucose,MAP,and serum T of rats in each group ($\bar{x}\pm SD$).

| Group（n=6） | Body weight（g） | | Random blood glucose(mmol/L) | | MAP(mmHg) | T(ng/ml) |
| --- | --- | --- | --- | --- | --- | --- |
|  | 9W | 21W | 9W | 21W |  |  |
| Control | 248.80±14.55 | 394.50±34.60 | 6.12±1.16 | 6.34±0.61 | 119.11±4.15 | 4.39±0.29 |
| Control+ICA | 250.90±12.03 | 400.70±29.01 | 6.26±0.82 | 6.80±1.13 | 117.41±3.75 | 4.43±0.34 |
| DM | 254.61±13.43 | 350.80±30.70 | 6.60±0.88 | 21.22±2.11^a^ | 122.36±4.72 | 3.94±0.31 |
| DM+ICA | 245.70±12.13 | 356.40±28.97 | 5.93±0.59 | 19.98±1.56^b^ | 116.20±4.44 | 4.07±0.27 |

Abbreviations:MAP, mean arterial pressure;T, testosterone;DM, diabetes mellitus

^a^p< 0.05 vs control group.

^b^p< 0.05 vs control+ICA group.

^c^p< 0.05 vs DM group.
