# Supplementary material for: Icariin inhibits hyperglycemia-induced cell death in penile cavernous tissue and improves erectile function in type 1 diabetic rats
Source: Sex Med. 2025 Mar 27;13(1):qfaf017. doi: 10.1093/sexmed/qfaf017 (PMC11950537; doi:10.1093/sexmed/qfaf017)
Supplement: Supplementary_Figure_qfaf017 [file supplementary_figure_qfaf017.docx]

**Supplementary Figure:**

Assessment of erectile function in the rats. (A) Representative curves of the MAP and ICPmax in each group of rats under 3 V and 5 V electrical stimulation. (B, C) Data represent the ratio of ICPmax/MAP in each group of rats under 3 V and 5 V electrical stimulation. & p < 0.05 vs. the control group. * p < 0.05 vs. the control + ICA group. # p < 0.05 vs. the DM group.
